# The interactions between autophagy and immune in the liver-adipose-ovary circuit of polycystic ovary syndrome

**DOI:** 10.3389/fimmu.2025.1733950

**Published:** 2026-01-20

**Authors:** Guofeng Nie, Muxuan Liu, Luxu Yang, Chanyu Li, Chenzhao Qu, Jing Wang, Juanjuan Mei, Yanlin Wang, Lei Han, Xinwei Zhang, Quanmin Wang

**Affiliations:** 1 The First School of Clinical Medicine, Binzhou Medical University Hospital, Binzhou Medical University, Binzhou, Shandong, China; 2 Department of Gynecology and Obstetrics, The First Affiliated Hospital of Chengdu Medical College, Chengdu, Sichuan, China; 3 Department of Reproductive Medicine, Binzhou Medical University Hospital, Binzhou, Shandong, China; 4 Department of Obstetrics and Gynecology, Binzhou Medical University Hospital, Binzhou, Shandong, China; 5 Department of Gynaecology and Obstetrics, Daping Hospital, Army Medical University, Chongqing, China

**Keywords:** adipose, autophagy, inflammatory, liver, polycystic ovary syndrome

## Abstract

Polycystic ovary syndrome (PCOS) is a common reproductive, endocrine, and metabolic disorder in women of reproductive age, characterized by hyperandrogenemia, insulin resistance, and ovulatory dysfunction. Autophagy, a key cellular homeostasis mechanism, closely interacts with immune-inflammatory responses to drive PCOS pathogenesis. This review highlights the “liver-adipose-ovary circuit”—a pathological network where the liver, adipose tissue, and ovaries crosstalk via autophagy dysregulation, chronic low-grade inflammation, and metabolic disturbances. Abnormal autophagy in adipose tissue induces insulin resistance and inflammatory cytokine release; hepatic autophagy impairment exacerbates non-alcoholic fatty liver disease (NAFLD) and hyperandrogenemia; ovarian autophagy dysfunction disrupts folliculogenesis. These organ-specific abnormalities form a self-reinforcing cycle that amplifies PCOS phenotypes. Clinical therapies targeting this circuit (e.g., quercetin, metformin) show promise by regulating autophagy, improving insulin sensitivity, and restoring reproductive-metabolic balance. Future research should clarify inter-organ molecular mediators and validate autophagy-targeted strategies to advance personalized PCOS treatment.

## 1 Introduction

### 1.1 Polycystic ovary syndrome

Polycystic ovary syndrome (PCOS), alternatively referred to as Stein-Leventhal syndrome, constitutes one of the most prevalent reproductive, endocrine, and metabolic disorders affecting women of reproductive age (1). This condition is potentially associated with both genetic and environmental determinants. PCOS is principally characterized by hyperandrogenemia (HA), insulin resistance (IR), ovulatory dysfunction, and polycystic alterations in the ovaries (2). The estimated global prevalence of PCOS ranges from approximately 5% to 15% (3).

### 1.2 Autophagy

Autophagy is a cellular self-digestion process that serves as a critical mechanism for maintaining intracellular homeostasis by degrading and recycling intracellular components (4). In response to various stress conditions, such as nutrient deprivation, oxidative stress, or mechanical stress, cells detect these stress signals, leading to the inhibition of mTOR and/or activation of AMPK and other core pathways, thereby initiating autophagy to ensure normal cellular survival and function (5). Autophagy plays a pivotal role in cellular immunity, affecting the development, differentiation, function, and homeostasis of immune cells, including T cells, B cells, and macrophages (6). Moreover, autophagy modulates inflammatory responses by influencing the production of pro-inflammatory cytokines, such as IL-1β and TNF-α, while cytokines like TNF-α, IL-1, IL-2, IL-6, and TGF-β can also induce autophagy (7). The interaction between autophagy and pro-inflammatory factors suggests a mechanism for balancing pro- and anti-inflammatory responses. This balance helps prevent excessive cellular and tissue damage.

Autophagy is integral to maintaining normal physiological functions. Disruption of autophagy can impair the immune functions of healthy organs and tissues, potentially leading to various diseases, including infections, autoimmune disorders, cancer, and metabolic disorders. In the context of metabolic disorders, dysregulated autophagy can exacerbate their progression, manifesting in conditions such as insulin resistance, diabetes, obesity, atherosclerosis, and osteoporosis. Furthermore, autophagy dysfunction is implicated in the pathophysiology of PCOS, where aberrant autophagy may constitute a critical pathway linking the dysfunction of multiple organ systems, thereby contributing to the onset and progression of the syndrome.

### 1.3 The liver-adipose-ovary circuit

Emerging evidence underscores that the reproductive and metabolic dysfunctions in PCOS are not isolated to a single organ but arise from intricate crosstalk among key metabolic and endocrine tissues. The “liver-adipose-ovary circuit” refers to the pathophysiological network formed during the pathogenesis of PCOS, where the liver, adipose tissue, and ovaries interact and exacerbate each other through mechanisms such as autophagy dysfunction, chronic low-grade inflammation, and metabolic disorders (such as insulin resistance and hyperandrogenism). This circuit describes a self-perpetuating, vicious cycle wherein dysfunction in one organ exacerbates abnormalities in the others, collectively driving and amplifying the core features of PCOS through shared mechanisms centered on autophagy dysregulation, chronic low-grade inflammation, and metabolic derangement.

## 2 Autophagy in adipose tissue and its role in immunity and inflammation in PCOS

Adipose tissue dysfunction is crucial in the etiology of PCOS (8). Adipose tissue functions not only as an energy reservoir but also as an active endocrine and immunoregulatory organ (3). In PCOS, abnormal adipose tissue autophagy leads to lipid metabolism disorders and increased release of inflammatory factors, directly or indirectly affecting systemic metabolism and reproductive function through related pathways.

### 2.1 The relationship between adipose tissue and autophagy

Mammals possess three primary types of adipocytes—white, beige, and brown, which are organized into distinct depots throughout the body (9). White adipose tissue (WAT) serves as a reservoir for fat storage during periods of energy surplus and facilitates the mobilization and release of fat during energy deficits. In animals, the two predominant forms of WAT are subcutaneous adipose tissue (SAT) and visceral adipose tissue (VAT) (10). Brown adipose tissue (BAT) is integral to weight regulation and metabolic control, as its activation enhances energy expenditure, mitigates obesity, reduces blood glucose and lipid levels, and secretes factors that influence both local and systemic energy metabolism. White adipocytes exhibit significant plasticity, enabling their trans-differentiation into beige adipocytes, which share numerous morphological and functional characteristics with brown adipocytes, particularly under stimuli such as exercise, cold exposure, and other factors (11).

Autophagy is essential for maintaining WAT homeostasis, primarily through three mechanisms: (1) Lipid regulation involves the modulation of fatty acid release through the autophagy-mediated degradation of lipid droplets (12). (2) Adipocyte differentiation is influenced by autophagy, which facilitates the transformation of preadipocytes into mature adipocytes. The deletion of autophagy-related genes, such as Atg7, has been shown to inhibit adipogenesis (13, 14). (3) In terms of stress adaptation, autophagy plays a crucial role in maintaining WAT homeostasis during nutritional fluctuations induced by endogenous or environmental stimuli. This is achieved by recycling damaged organelles, thereby underscoring autophagy’s dynamic homeostatic regulatory properties (15). Furthermore, autophagy positively impacts BAT by preserving mitochondrial quality and thermogenesis, while also promoting the differentiation of brown adipocytes (16). Impairments in autophagy can result in BAT dysfunction, where defective mitophagy leads to the accumulation of reactive oxygen species (ROS), activation of inflammatory pathways, and subsequent inflammatory responses (17).

### 2.2 The relationship between adipose tissue autophagy and PCOS immunity and inflammation

#### 2.2.1 Adipocyte hypertrophy promotes insulin resistance in PCOS

The volume of VAT in patients with PCOS is approximately 30% greater than in individuals with a BMI, with an increase in adipocyte diameter of about 20% (18–20). This study corroborates that adipose tissue in PCOS predominantly undergoes hypertrophy rather than hyperplasia. The enlargement of adipocytes in PCOS patients is associated with decreased insulin sensitivity.

The perigonadal adipose tissue, a component of the visceral fat depot that includes the fat surrounding the ovaries and uterus, functions not merely as an energy reservoir but as a dynamic endocrine and immune organ capable of regulating ovarian function (21). Hypertrophy of perigonadal adipose tissue adipocytes triggers a cascade of pathophysiological changes. In patients with PCOS, an increase in adipocyte volume is associated with diminished insulin sensitivity. Initially, the activation of NOD-like receptor family pyrin domain-containing 3 (NLRP3) inflammasomes occurs within hypertrophied adipocytes during obesity, facilitating the maturation of IL-1β and IL-18, thereby intensifying inflammatory responses. Concurrently, the expression of autophagy-related genes, such as LAMP1, LAMP2, and Atg5, is downregulated, while the expression of genes associated with inflammation, including MCP-1, IL-6, and IL-1β, is upregulated. The hypertrophied adipocytes contribute to collagen deposition and fibrosis, resulting in the remodeling of adipose tissue and the onset of insulin resistance. Furthermore, these adipocytes, with compromised autophagic processes, release elevated levels of free fatty acids (FFAs) and pro-inflammatory mediators such as TNF-α, IL-6, and MCP-1, which further promote the development of insulin resistance, while simultaneously reducing the secretion of anti-inflammatory factors like adiponectin (22). TNF-α is known to activate inflammatory signaling pathways, including JNK and IKKβ, which in turn stimulate the serine phosphorylation of insulin receptor substrate 1 (IRS-1), leading to decreased expression of IRS-1 and glucose transporter type 4 (GLUT-4). Inhibiting their normal tyrosine phosphorylation weakens insulin-stimulated glucose uptake capacity, blocking insulin signaling and resulting in insulin resistance (23).

#### 2.2.2 Role of adipokines in PCOS

Adiponectin plays a pivotal role in regulating total body fat by modulating energy balance and exhibits insulin-sensitizing properties. In patients with PCOS, there is a dysregulation of adipokines, characterized by decreased adiponectin levels, which are associated with abdominal obesity and hyperandrogenism, whereas leptin levels are elevated (24). Studies suggest that adiponectin enhances insulin sensitivity by activating autophagy flux in skeletal muscle, facilitating the clearance of misfolded proteins, and mitigating endoplasmic reticulum stress (25). In mouse models of obesity and insulin resistance induced by a high-fat diet, adiponectin supplementation has been shown to stimulate autophagy and reduce oxidative stress, thereby enhancing insulin sensitivity and ameliorating insulin resistance (26).

Leptin, predominantly secreted by adipocytes in WAT, is integral to energy metabolism, appetite regulation, and immune function (27). Under normal physiological conditions, leptin upregulates insulin-like growth factor-binding protein-2 (IGFBP-2) expression and activates the AMPK pathway, thereby promoting glucose uptake and utilization, increasing fatty acid oxidation, and enhancing insulin sensitivity. Furthermore, leptin mitigates inflammation induced by endoplasmic reticulum stress by attenuating Atf4-Atg5-mediated autophagy in adipocytes (28). Experimental studies in animals indicate that leptin activates autophagy-related PI3K/Akt/mTOR signaling pathways, thereby enhancing proliferation and inhibiting apoptosis in goose granulosa cells, which underscores leptin’s direct involvement in the regulation of the reproductive system (29).

#### 2.2.3 Adipose tissue macrophages influence PCOS through inflammation

In the context of immune inflammation, adipose tissue macrophages (ATMs) are crucial. Macrophages can differentiate into either pro-inflammatory (M1) or anti-inflammatory (M2) phenotypes, contingent upon the signals they receive and their microenvironment. The equilibrium between M1 and M2 macrophages is critical in determining physiological and pathological outcomes (30). In patients with PCOS, there is an increased proportion of M1 macrophages and a decreased proportion of M2 macrophages within VAT. This imbalance in immune cell polarization leads to the excessive secretion of pro-inflammatory cytokines such as TNF-α, IL-6, IL-1β, IL-17, and IFN-γ, resulting in the formation of “inflammatory foci” within the adipose tissue. Inflammatory factors can adversely affect insulin-related signaling pathways in adipocytes, diminishing their sensitivity to insulin stimulation and ultimately contributing to insulin resistance. In adipose tissue, M1 macrophages inhibit autophagy by secreting pro-inflammatory cytokines, including TNF-α, IL-6, and IL-8 (31, 32). TNF-α activates the NF-κB and JNK signaling pathways, suppresses autophagy-promoting factors such as AMPK, and concurrently enhances mTOR activity, thereby obstructing autophagy. The modulation of autophagy in adipose tissue plays a significant role in the initiation and progression of inflammation. During obesity, autophagy in adipocytes is upregulated in a pro-inflammatory manner. However, excessive autophagy can result in mitochondrial dysfunction and the accumulation of ROS, which in turn activate NF-κB signaling and induce inflammation (33).

## 3 Liver autophagy in PCOS and its role in immunity and inflammation

The liver, as a pivotal organ for metabolic and immune regulation, can directly or indirectly influence ovarian function through mechanisms such as lipid metabolism disorders, insulin resistance, inflammatory responses, and autophagy dysregulation, contribute to the phenotype of PCOS (34, 35). Dysfunctional hepatic autophagy can disrupt metabolic, immune, and inflammatory processes, potentially leading to systemic metabolic disorders and functional abnormalities. This dysfunction is closely linked to the co-occurrence of non-alcoholic fatty liver disease (NAFLD) in PCOS.

### 3.1 The relationship between hepatic autophagy function and the pathophysiology of PCOS

The liver, as the body’s metabolic hub, is integral to processes such as nutrient synthesis and breakdown, hormone inactivation, and biotransformation (36). Within these processes, hepatic autophagy is vital for maintaining metabolic homeostasis. Autophagy serves as a protective mechanism by efficiently removing damaged organelles and proteins, thereby safeguarding hepatocytes from further damage (37). Conversely, aberrant autophagy responses can induce hepatocyte death and liver dysfunction, a phenomenon observed in various hepatic pathologies, including viral hepatitis, NAFLD, liver fibrosis, and hepatocellular carcinoma (38). Additionally, the liver plays a significant role in immune modulation (39). Hepatic autophagy is intricately connected to the activity of immune cells within the liver, such as Kupffer cells and T cells.

NAFLD is a chronic hepatic disorder characterized by the accumulation of fat in the liver and is frequently associated with metabolic conditions such as obesity, insulin resistance, and type 2 diabetes (40). The prevalence of NAFLD is notably higher among women with PCOS, and conversely, women with NAFLD are more likely to exhibit PCOS. This reciprocal relationship suggests a strong interconnection between the two conditions, potentially establishing a bidirectional vicious cycle (41).

The impaired autophagy suppression in NAFLD patients contributes to lipid accumulation, the release of pro-inflammatory factors, and the generation of reactive oxygen species, which collectively can lead to excessive activation of the JNK pathway in hepatocytes, culminating in hepatic insulin resistance (42). The resultant state of hyperinsulinemia associated with hepatic insulin resistance can further enhance Akt/mTOR signaling, inhibit autophagy, and facilitate the progression of hepatic steatosis (43, 44).

**Figure 1:**
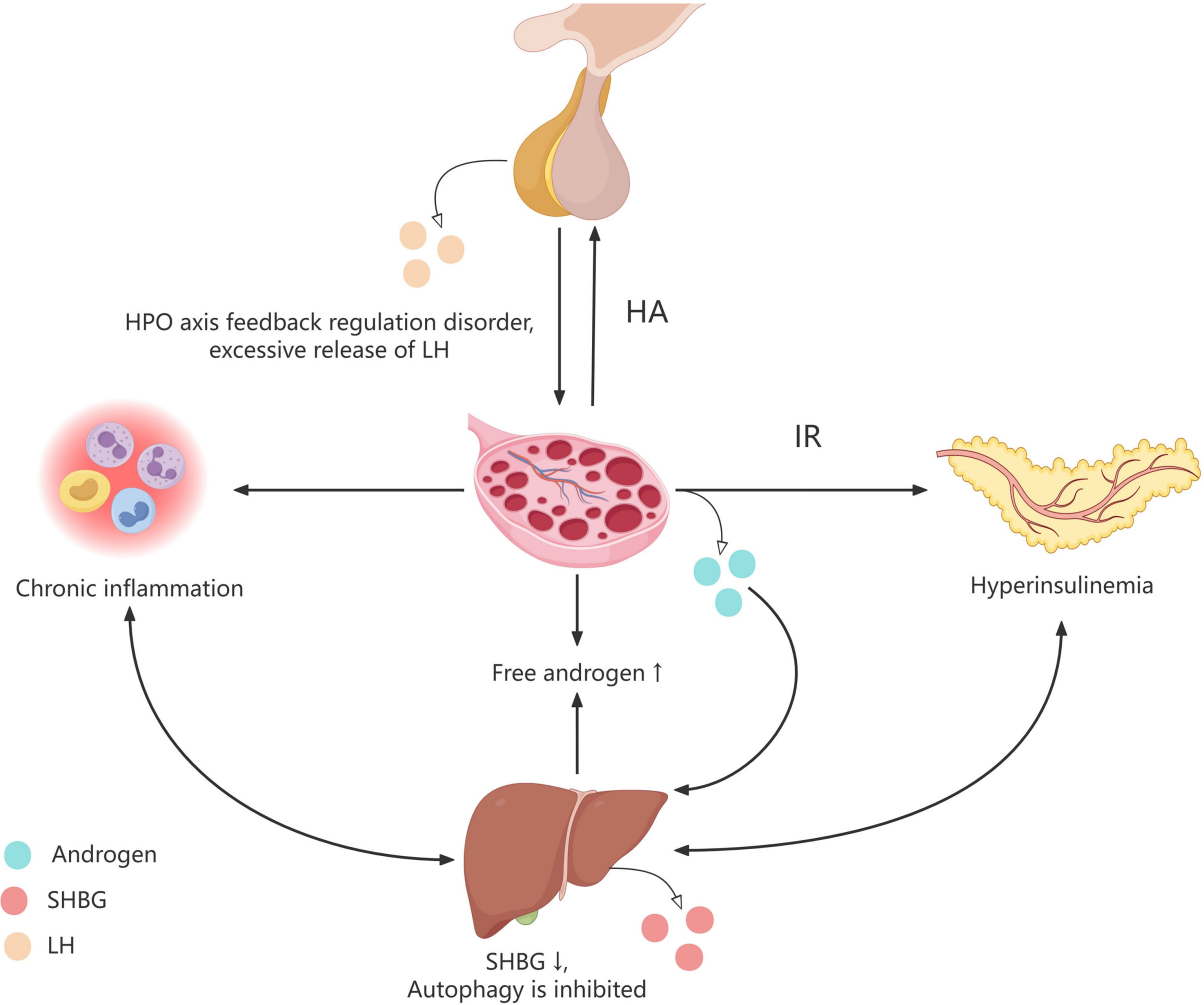
Metabolic characteristics of PCOS patients. PCOS women are characterized by long-term chronic inflammation, insulin resistance, and hyperandrogenism. Elevated levels of free androgens in the circulation disrupt the negative feedback regulation mechanism of the hypothalamic pituitary ovarian axis, leading to disrupted pulse release of luteinizing hormone and excessive release of luteinizing hormone, promoting the synthesis and secretion of androgens in PCOS patients. The elevated levels of androgens in the circulation cause a decrease in insulin sensitivity in various tissues of the body, resulting in hyperinsulinemia. In addition, long-term chronic inflammation, hyperinsulinemia, and high circulating hormone levels inhibit the production of SHBG in the liver, suppress autophagy levels in liver related cells, and further lead to an increase in circulating free androgen levels, insulin resistance, and chronic inflammatory response. HPO, hypothalamic-pituitary-ovarian; HA, Hyperandrogenemia; IR, Insulin resistance; SHBG, Sex hormone-binding globulin.

Moreover, research indicates that hepatic insulin resistance downregulates the activity of the sex hormone-binding globulin (SHBG) gene promoter, thereby inhibiting SHBG gene transcription and reducing hepatic SHBG production. Since SHBG serves as the primary binding protein for androgens, decreased levels result in increased circulating free androgens, ultimately leading to hyperandrogenemia (45). Elevated circulating androgens form activated androgen receptor-ligand complexes that translocate to the nucleus, where they bind to androgen response elements (AREs) within the SHBG promoter region and recruit co-repressors to inhibit SHBG gene transcription. This mechanism allows androgens to directly downregulate SHBG gene expression, thereby reducing SHBG mRNA production and further inhibiting hepatic SHBG synthesis (46–48). Consequently, androgen levels continue to rise, establishing a vicious positive feedback loop that exacerbates androgen-related symptoms in patients with PCOS. Furthermore, elevated circulating free androgen levels disrupt the negative feedback regulation of the hypothalamic-pituitary-ovarian (HPO) axis (**Figure 1**). This condition diminishes the feedback inhibition of luteinizing hormone (LH), resulting in disrupted gonadotropin-releasing hormone (GnRH) pulsatility and excessive LH release, which further exacerbates abnormal androgen levels (34).

These findings suggest that abnormal liver autophagy may impair ovarian function via insulin resistance and hyperandrogenemia, contributing to ovarian metabolic disorders and potentially playing a role in PCOS pathophysiology.

### 3.2 Abnormal hepatic autophagy contributes to immune-inflammatory dysregulation in PCOS

Kupffer cells, the resident macrophages of the liver, play a crucial role in the hepatic immune response. Insulin resistance leads to excessive activation of the mTOR pathway in hepatocytes, inhibiting autophagy (43, 44). Impaired autophagy hinders clearance of damaged mitochondria and lipid droplets (49), causing lipid accumulation. This induces lipotoxicity, activating Kupffer cells via TLR4 to produce ROS and secrete TNF-α and other pro-inflammatory cytokines, promoting polarization toward the M1 phenotype and resulting in liver injury and fibrosis (50). ROS and mtDNA release from damaged mitochondria activate the NLRP3 inflammasome, increasing secretion of IL-1β and IL-18 and exacerbating inflammation (51). Deletion of Atg5 in Kupffer cells of high-fat diet mice induces M1 polarization and inflammatory mediator secretion (52). These factors can disseminate systemically, triggering systemic inflammation (53).

PCOS prevalence is linked to inflammatory factors that infiltrate ovarian tissue, disrupting follicular development and ovulation (54). Dysregulated hepatic autophagy disrupts the M1/M2 macrophage balance, releasing inflammatory mediators that impair ovarian physiology, associated with PCOS pathophysiology. Chronic inflammation worsens insulin resistance and hyperandrogenemia, creating a vicious cycle.

Impaired hepatic autophagy correlates with pro-inflammatory T cells. Th1 cells secrete IL-2 and IFN-γ to enhance cellular immunity, while Th2 cells secrete IL-4 for humoral immunity (55). In high-fat diet mice, reduced Th2 and increased CD8+ T, Th1, and B cells link to NAFLD with fibrosis, involving impaired autophagy, ER stress, and NLRP3 activation (56). PCOS patients show a Th1-dominant response, with elevated Th1/Th2 ratio correlating with higher BMI, indicating chronic low-grade inflammation (57). Th1 dominance may exacerbate insulin resistance and hyperandrogenism. The shared Th1/Th2 shift in NAFLD and PCOS suggests autophagy deficiencies and T-cell dysfunction contribute to their common pathophysiology.

## 4 Ovarian autophagy in PCOS and its role in immunity and inflammation

The ovaries, as integral components of the female reproductive system, are responsible for the production of oocytes and sex hormones, a process regulated by gonadotropins. The fundamental functional units within the ovaries are the ovarian follicles, which are essential for hormone production and oocyte development (58). Autophagy, a critical mechanism for maintaining cellular homeostasis, is involved in several vital physiological processes, including follicular recruitment, selection of the dominant follicle, and the clearance of follicular atresia, all of which are essential for normal ovarian function (59, 60).

### 4.1 The role of autophagy in the ovary

In the context of PCOS, this intricately regulated network experiences multidimensional dysregulation. During the typical development of ovarian follicles in mammals, the autophagic activity within follicular cells is vital for preserving oocyte quality, and any disruption in this process may result in female infertility (61).

On the one hand, autophagy plays a significant role in the regulation of follicular atresia. A process wherein the number of follicles within the ovaries diminishes with age, with the majority undergoing natural atresia at various developmental stages. This process is modulated by both apoptosis and autophagy. Research findings suggest that electron microscopy has identified a significant presence of lysosomes and autophagosomes within the cytoplasmic matrix of rat oocytes, indicating that active autophagy may play a role in oocyte development and maturation. Hormonal regulation also appears to influence autophagic processes. For example, under conditions of oxidative stress, melatonin facilitates the survival of granulosa cells (GCs) by inhibiting autophagy during follicular atresia, achieved through the suppression of the transcription factor forkhead box O1 (FOXO1) (62). Treatment with follicle-stimulating hormone (FSH) has been shown to suppress autophagy in GCs (63), suggesting that FSH may protect mouse granulosa cells from oxidative damage by inhibiting mitophagy (64). Furthermore, FSH inhibits apoptosis in GCs via the PI3K/Akt/mTOR signaling pathway and FOX gene transduction in human ovarian GCs, which is essential for maintaining follicular atresia and promoting GCs proliferation (65).

On the other hand, autophagy exerts an influence on ovarian reserve, thereby affecting follicular and oocyte development. In instances of follicular depletion, the anti-Müllerian hormone (AMH), synthesized by GCs of early-stage developing follicles, can safeguard the immature follicle reserve by inhibiting autophagy within the ovary through the suppression of FOXO3/FOXO3A phosphorylation, which otherwise leads to the activation of immature follicles (66). The Atg7 gene, a pivotal autophagy-related gene essential for autophagosome formation, maintains stable expression levels throughout all stages of oogenesis. Mice deficient in the Atg7 gene typically exhibit reduced litter sizes and experience a gradual decline in fertility. These mice display a significant reduction in the number of germ cells and primordial follicles, with numerous follicles demonstrating structural abnormalities or functional loss. This evidence suggests that autophagy is crucial for germ cell survival (67). Furthermore, studies have indicated that oxidized low-density lipoprotein (OxLDL) may exacerbate fertility challenges in obese women by inducing autophagic cell death in ovarian granulosa cells. These research findings reveal that the autophagy process within follicles and their overall metabolic status significantly influence follicular development, with these effects carrying profound implications for reproductive health.

### 4.2 The role of autophagy in immune dysregulation and inflammation in PCOS ovaries

Macrophages, dendritic cells (DCs), neutrophils, eosinophils, mast cells, B cells, T cells, and natural killer (NK) cells constitute the immune cell population present in the ovaries (68). These ovarian immune cells perform a variety of functions, including phagocytosis and antigen presentation, tissue remodeling through proteolytic enzyme activity, and the secretion of soluble mediators such as cytokines, chemokines, and growth factors (69). Among these, macrophages are the most prevalent immune cells within ovarian tissue and are integral to maintaining the stability of the ovarian microenvironment (70). In patients with PCOS and corresponding animal models, there is an observed increase in the number of M1 macrophages in both peripheral blood and ovarian tissue, accompanied by an elevated M1/M2 macrophage ratio and increased levels of C-reactive protein and proinflammatory factors (71). Studies have demonstrated that in the context of PCOS, macrophage-derived proinflammatory factors, such as IL-6, IL-18, and TNF-α, contribute to dysregulated autophagy in granulosa cells. Patients with PCOS exhibit upregulated expression of autophagy-related genes ATG5 and ATG7 in granulosa cells, along with an increased LC3II/LC3I ratio. This dysregulation may impair the processes of follicular growth, maturation, and atresia, thereby leading to disorders in follicular development and adversely affecting female fertility (59, 60).

Ovarian ovulation is characterized by localized damage to the follicle wall, followed by a healing process that constitutes an acute aseptic inflammatory response. Within the ovary, DCs represent the primary population of bone marrow-derived immune cells surrounding mature oocytes. As pivotal immune regulators, DCs facilitate follicle rupture and oocyte release by detecting inflammatory signals and modulating cytokine release. Autophagy plays a dual regulatory role in this process: it inhibits the immunogenic maturation of DCs, for instance, by decreasing MHC-II expression, while simultaneously promoting their tolerogenic maturation, such as through the induction of Treg differentiation (72). Studies have identified that abnormalities in DC maturation and cytokine production may contribute to atypical oocyte development in patients with PCOS (73). In the context of PCOS, hyperandrogenemia and insulin resistance may disrupt the autophagic equilibrium in DCs, resulting in impaired immune tolerance and the aberrant release of pro-inflammatory cytokines, including IL-1β and TNF-α. This disruption subsequently fosters chronic ovarian inflammation and ovulatory dysfunction.

In ovarian tissue affected by PCOS, insulin resistance and hyperandrogenemia have been identified as factors associated with low-grade inflammation. Recent studies have illustrated that abnormal inflammatory processes can disrupt normal ovarian follicular dynamics, resulting in compromised oocyte quality, anovulation, and subsequent infertility (74). High Mobility Group Box 1 (HMGB1), a critical inflammatory mediator, is found at significantly elevated levels in both the blood circulation and follicular fluid of women with PCOS, in conjunction with insulin resistance (75, 76). HMGB1 exacerbates inflammation and insulin resistance via the NF-κB signaling pathway and directly induces autophagy in ovarian granulosa cells. This is evidenced by increased LC3B-II/I ratios and ATG7 levels, along with decreased SQSTM1 levels. Such excessive autophagy leads to a reduction in granulosa cell numbers and disrupts insulin signaling pathways, including AKT phosphorylation, GLUT4 translocation, and glucose uptake, thereby impairing ovarian function. Research has demonstrated that these pathological effects can be reversed by inhibiting HMGB1-mediated autophagy (77).

Insulin-like growth factor-1 (IGF-1) is a hormone implicated in the induction of inflammatory cytokine production. Elevated IGF-1 levels have been documented in PCOS and may be associated with autophagy (78). Recent research involving zebrafish ovaries suggests that locally synthesized IGF-1 within the ovaries facilitates the growth and development of primary follicles (79). IGF-1 has been shown to regulate cellular autophagy via the PI3K/mTOR signaling pathway (80). Targeting IGF-1 to modulate the initiation of autophagy through the mTOR pathway may enhance therapeutic outcomes for patients with PCOS and early-stage endometrial cancer (81).

## 5 Autophagy-mediated immune regulation of the liver-adipose-ovary circuit in PCOS

**Figure 2:**
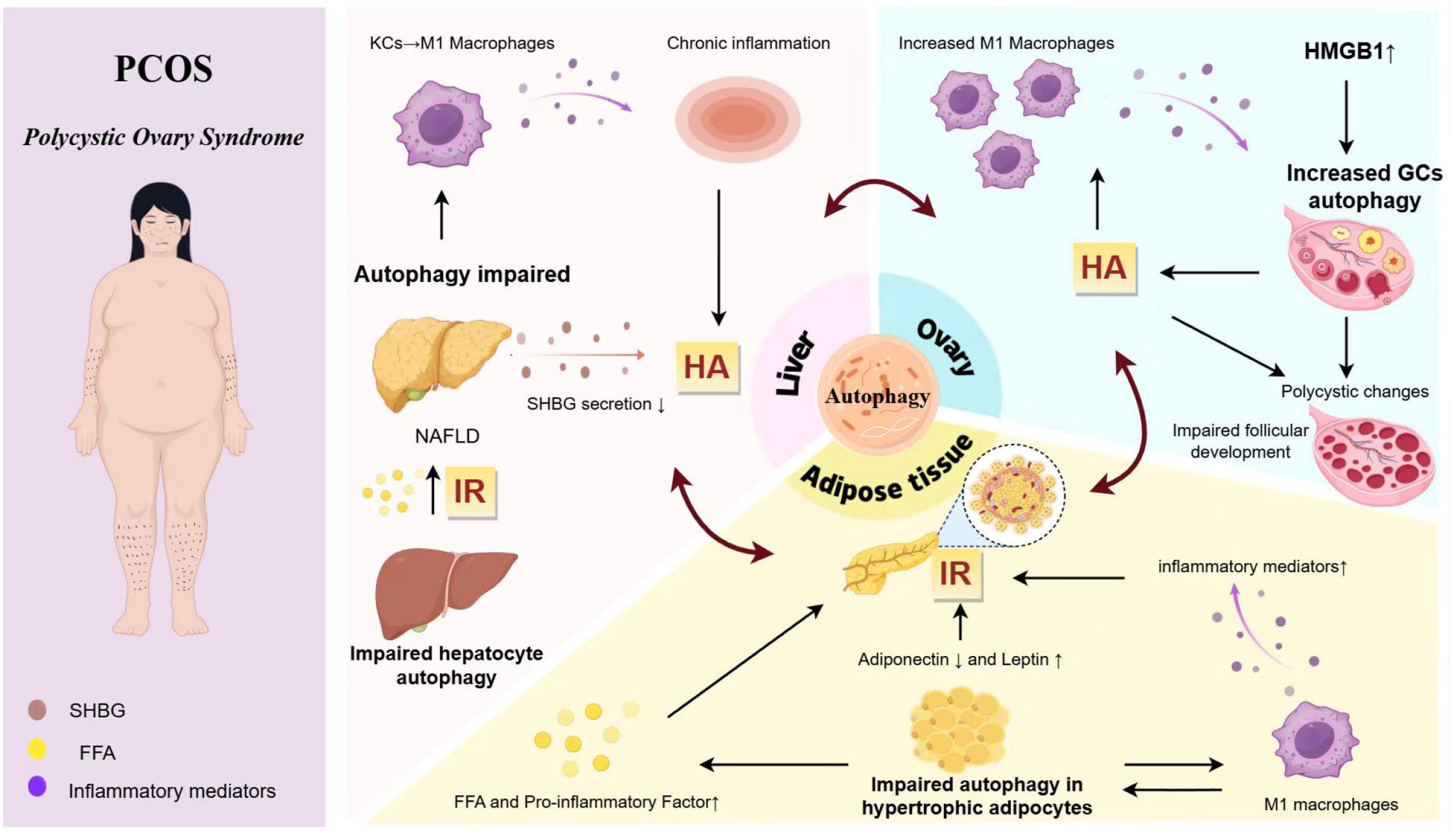
Autophagy-mediated immune regulation of the liver-adipose-ovary circuit in PCOS. Dysfunctional autophagy in adipose tissue drives IR and systemic inflammation, which interacts reciprocally with HA stemming from hepatic and ovarian tissues with impaired autophagy and ovarian tissues, forming a vicious cycle. a) Hypertrophy of autophagy-impaired adipocytes and activation of M1 macrophages mutually reinforce each other, releasing adipokines, free fatty acids FFAs, and pro-inflammatory factors, thereby inducing IR. b) The release of FFAs and pro-inflammatory factors due to autophagy dysregulation triggers non-alcoholic NAFLD. This process activates M1 macrophages, promoting systemic chronic inflammation and reducing SHBG synthesis, ultimately leading to HA. c) HMGB1 and HA disrupt ovarian cellular autophagy, inducing perigonadal inflammatory responses and contributing to the polycystic transformation of the ovaries. IR, insulin resistance; HA, hyperandrogenemia; KCs, Kupffer cells; FFA, free fatty acids; HMGB1, high mobility group beta-1; GCs, granulosa cells; NAFLD, non-alcoholic fatty liver disease. SHBG, Sex hormone-binding globulin.

The pathogenesis of PCOS involves complex interactions across multiple organ systems, with autophagy-mediated immune-inflammatory regulation playing a pivotal role. Dysregulations in autophagy-related immune-inflammatory processes are evident in the liver, adipose tissue, and ovaries, which interact and collectively contribute to the multi-organ dysfunction associated with the autophagy-immune signaling network in PCOS (**Figure 2**).

The hypertrophy of adipose cells, coupled with impaired autophagy, facilitates the release of FFAs, infiltration of macrophages, and secretion of pro-inflammatory factors. These processes collectively inhibit insulin signaling pathways, thereby inducing insulin resistance (82–85). Insulin resistance adversely impacts the liver, resulting in reduced insulin sensitivity and activation of the mTOR signaling pathway, which further suppresses autophagy (86). The excessive influx of FFAs contributes to triglyceride accumulation, precipitating NAFLD and diminishing adiponectin levels, thereby exacerbating hepatic steatosis. Simultaneously, hyperinsulinemia inhibits the synthesis of SHBG, leading to increased levels of free androgens and aggravating hyperandrogenism (87). Both systemic and local ovarian insulin resistance augment ovarian CYP17 activity, promoting androgen synthesis and further elevating androgen levels by stimulating pituitary LH release. Elevated androgen levels subsequently inhibit the development of dominant follicles. When combined with localized ovarian signaling abnormalities induced by insulin resistance, impaired glucose metabolism, and abnormal cell proliferation, these factors contribute to the development of polycystic ovarian-like alterations and anovulation (88–90). Elevated androgen levels and excessive inflammatory mediators, further influenced by genetic predispositions and lifestyle factors, contribute to adipocyte hypertrophy and proliferation. This exacerbates insulin resistance and inflammation, leading to complications such as metabolic syndrome, thereby perpetuating the pathological cycle of PCOS (91–93).

Impaired autophagy in the hepatocytes of PCOS patients hinders the clearance of damaged mitochondria, disrupting mitochondrial fatty acid oxidation and increasing FFA release. These FFAs are transported to adipose tissue via the portal vein, where they activate PKC to phosphorylate IRS1.This phosphorylation event inhibits the PI3K/Akt signaling pathway, which is essential for regulating lipid autophagy, thus exacerbating autophagy dysfunction in adipocytes and contributing to insulin resistance. The resultant insulin resistance in adipose tissue further aggravates hepatic autophagy defects, perpetuating a detrimental cycle between adipose tissue and the liver (94). Concurrently, impaired hepatic autophagy diminishes the liver’s capacity to clear steroids, resulting in elevated circulating androgen levels, particularly DHT. Elevated DHT levels enhance pro-inflammatory responses in adipocytes, exacerbate immune dysregulation within adipose tissue—such as by suppressing eosinophil activity—and induce aberrant autophagy pathways in ovarian granulosa cells. These alterations impair steroidogenesis and adversely affect follicular development (95). Furthermore, aberrant hepatic autophagy induces a phenotypic shift in Kupffer cells from an M2 anti-inflammatory state to an M1 pro-inflammatory state, leading to the activation of NLRP3 inflammasomes and the subsequent release of cytokines such as IL-1β and TNF-α (96–100). These systemic inflammatory mediators exert direct cytotoxic effects on ovarian granulosa cells, inhibit aromatase activity, activate the NF-κB signaling pathway to trigger apoptosis, and disrupt follicular development and ovulation, thereby sustaining the pathological condition of PCOS (101–103). As the central organ of reproductive function, the ovaries perpetuate the pathological cycle of PCOS through local dysregulation of autophagy and immune responses. This dysregulation influences adipose tissue and hepatic function via aberrant hormone secretion, inflammatory signaling, and the regulation of steroid synthesis. In PCOS, abnormal autophagy in ovarian granulosa cells results in elevated androgen secretion. These circulating androgens impact adipose tissue by altering the macrophage phenotypic balance within adipocytes and promoting the release of inflammatory cytokines, which further compromise adipocyte autophagy function (104).

Concurrently, androgens contribute to the development of insulin resistance, which subsequently aggravates hepatic metabolic abnormalities. These hepatic metabolic disturbances further stimulate ovarian androgen synthesis, establishing a self-perpetuating cycle of “hyperandrogenism-insulin resistance-autophagy defects” (105, 106). Moreover, PCOS is characterized by an increased presence of pro-inflammatory macrophages within the ovaries. The inflammatory cytokines secreted by these macrophages not only worsen the autophagy dysfunction within the ovaries but also disseminate to influence adipose tissue and the liver, thereby promoting adipocyte hypertrophy and enhancing pro-inflammatory cell infiltration in adipose tissue. Concurrently, these cytokines modify the phenotype of immune cells in the liver and diminish hepatic immune tolerance. Furthermore, inflammatory mediators abnormally released due to impaired autophagy in ovarian granulosa cells exacerbate local insulin resistance within the ovary. These mediators subsequently enter systemic circulation, triggering inflammatory responses in adipose tissue and the liver, which leads to further deterioration of adipose and hepatic function. Furthermore, ovarian estrogen synthesis is dependent on lipids, and aberrant autophagy in granulosa cells disrupts lipid utilization, resulting in diminished estrogen production (107). Estrogen plays a protective role in the liver by alleviating hepatic damage and modulating metabolic processes. A deficiency in estrogen exacerbates liver dysfunction, thereby contributing to the formation of a pathological network characteristic of PCOS. This network involves the coordinated interaction of multiple organs, including the ovaries, adipose tissue, and liver (108).

**Figure 3:**
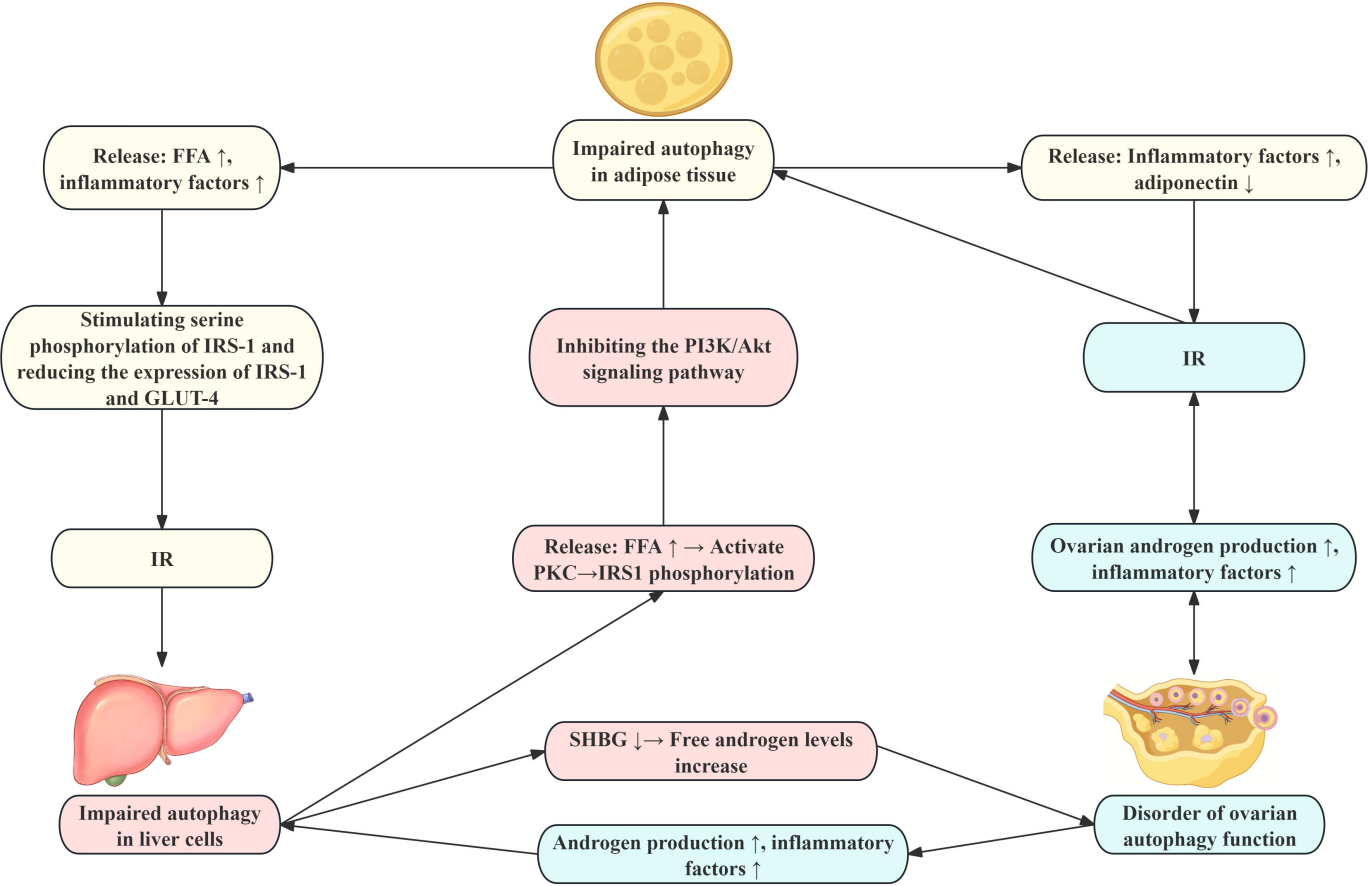
Pathophysiological changes in the liver-adipose-ovary circuit in PCOS. This figure depicts the interactions and related mechanisms among the various organs in the liver-adipose-ovary circuit in patients with PCOS. IR, insulin resistance; HA, hyperandrogenemia; KCs, Kupffer cells; FFA, free fatty acids; IRS-1, Insulin receptor substrate 1; GLUT-4, Glucose transporter 4; SHBG, Sex hormone-binding globulin.

In summary, autophagy forms an interdependent pathological network in the liver, adipose tissue, and ovaries of PCOS: Abnormal autophagy in adipose tissue leads to inflammatory cytokine release and insulin resistance, which in turn affects hepatic autophagy function via the circulatory system, exacerbating lipid deposition and pro-inflammatory polarization of Kupffer cells; Defective hepatic autophagy further exacerbates ovarian autophagy dysfunction via impaired androgen metabolism and systemic inflammation, causing granulosa cell dysfunction and abnormal follicular development; Concurrently, ovarian autophagy dysregulation promotes macrophage infiltration in adipose tissue and hepatic lipotoxicity through feedback mechanisms involving hyperandrogenism and inflammatory mediators like HMGB1. This establishes a “liver-adipose-ovary” autophagy-immunity-metabolism cycle that collectively leading to reproductive dysfunction and metabolic abnormalities in PCOS (**Figure 3**).

## 6 Clinical treatment related to the PCOS liver-adipose-ovary circuit

Caloric restriction and regular exercise have been shown to activate the AMPK pathway and inhibit mTOR signaling, thereby promoting protective autophagy in adipose tissue and liver. This improves insulin sensitivity and reduces hepatic lipid accumulation (109–111). The normalization of autophagy helps break the vicious cycle of metabolic disorders and chronic inflammation.

As an AMPK activator, metformin enhances autophagy in the liver and adipose tissue, improving insulin resistance. Studies indicate it can attenuate HMGB1-mediated excessive autophagy in ovarian granulosa cells, protecting follicular development (112, 113). Its combination with berberine synergistically regulates the AMPK/AKT/mTOR pathway, more effectively restoring autophagic homeostasis in the ovaries and metabolic tissues of PCOS rats (114). Beyond improving metabolic parameters, GLP-1 RAs activate PKA and AMPK pathways to promote lipophagy in white adipose tissue and suppress inflammation-associated autophagic dysregulation in the liver and ovaries (115–117). Their weight loss and visceral fat reduction effects are partly attributed to autophagy-mediated adipose tissue remodeling.

Functioning as a SIRT1 activator, quercetin enhances autophagic flux to improve insulin sensitivity in adipose tissue. In the ovaries, it inhibits androgen-induced autophagic disturbances, thereby supporting normal follicular development (118). Melatonin mitigates oxidative damage to ovarian granulosa cells by suppressing FOXO1-mediated excessive autophagy. Simultaneously, it promotes protective autophagy in the liver and adipose tissue, improving systemic metabolic status (119). Traditional Chinese Medicine formulations often modulate autophagy-related pathways (e.g., PI3K/AKT/mTOR) through multi-target actions to ameliorate metabolic and reproductive abnormalities in PCOS (120). IDO inhibitors (e.g., 1-methyltryptophan) may alleviate ovarian local inflammation and autophagic imbalance by regulating the tryptophan metabolism-autophagy axis (121).

Current PCOS treatments directly or indirectly target autophagy pathways, aiming to restore autophagic homeostasis within the “adipose-liver-ovary” circuit and improve metabolic and reproductive phenotypes. Future research should further elucidate the precise tissue-specific mechanisms of autophagy regulation by different therapies and explore individualized treatment strategies based on autophagy-related biomarkers, providing new avenues for the precision management of PCOS.

## 7 Current gaps and controversies

Autophagy plays a pivotal yet complex role in the PCOS “liver-adipose-ovary” circuit, with research facing three major challenges.

Autophagy exhibits context-dependent duality, serving protective roles in metabolic homeostasis while contributing to inflammation and insulin resistance when dysregulated, as seen in defective mitophagy (23, 32). Future work should focus on autophagic flux dynamics and pathway crosstalk rather than static activity measures. Animal model heterogeneity limits translational relevance, with DHEA-induced models showing suppressed ovarian autophagy (104) and high-fat-diet models indicating enhanced adipose autophagy (12). More physiologically relevant models and clinical validation are needed.

Clinical biomarker development remains challenging due to poor correlation between circulating markers (e.g., p62/SQSTM1, ATG5/ATG7) and tissue-specific autophagic activity, dynamic process variability, and patient heterogeneity. Advancing exosome-based markers, multi-parameter models, and treatment-response predictors will be crucial for precision PCOS management.

## 8 Summary and prospect

Future research should aim to clarify how autophagy and immune inflammation interact within the “liver-adipose-ovary” network in PCOS. Although dysregulated autophagy is linked to metabolic and reproductive issues, its varying roles in different tissues are not fully understood. Research should identify molecular mediators of inter-organ communication, using advanced technologies like single-cell sequencing to find markers for PCOS subtypes. Additionally, targeting autophagy pathways for treatment shows promise but needs validation in animal and clinical studies to ensure safety and effectiveness in regulating autophagy, improving insulin resistance, and restoring ovarian function.

In-depth exploration of theca cell function and regulation, which is the core site of ovarian androgen synthesis, has revealed that their dysfunction causes hyperandrogenemia in PCOS patients. Moreover, it forms a vicious cycle with inflammatory signals from visceral fat and insulin resistance, thereby driving reproductive and metabolic abnormalities. The underlying mechanisms remain to be fully elucidated (122, 123). At the same time, breakthroughs in the research on the autophagy of immune cells and mitochondrial phagocytosis disorders in PCOS women will reveal the intrinsic association between oxidative stress, inflammation and immune autophagy, providing new intervention targets and treatment strategies to break the vicious cycle of chronic inflammation and insulin resistance and improve the reproductive and metabolic abnormalities of PCOS (124, 125).

The key challenges include PCOS’s high variability, limitations in autophagy monitoring, and differences between animal models and human conditions. The absence of reliable, non-invasive biomarkers for real-time autophagy assessment complicates evaluating clinical interventions. Autophagy’s varying effects across tissues and disease stages make its regulation difficult in therapy design. Future efforts should focus on interdisciplinary collaboration to integrate multi-omics data with clinical profiles, develop a molecular subtyping system for PCOS, and conduct long-term studies. This approach will clarify autophagy’s role in PCOS prevention and management, advancing personalized treatment strategies.
